# New Denoising Method for Lidar Signal by the WT-VMD Joint Algorithm

**DOI:** 10.3390/s22165978

**Published:** 2022-08-10

**Authors:** Zhenzhu Wang, Hongbo Ding, Bangxin Wang, Dong Liu

**Affiliations:** 1 Key Laboratory of Atmospheric Optics, Anhui Institute of Optics and Fine Mechanics, Hefei Institutes of Physical Science, Chinese Academy of Sciences, Hefei 230031, China; 2 Science Island Branch of Graduate School, University of Science and Technology of China, Hefei 230026, China; 3 Advanced Laser Technology Laboratory of Anhui Province, Hefei 230037, China

**Keywords:** lidar, denoising, WT, EMD, VMD, SSA, WT-VMD

## Abstract

Light detection and ranging (LIDAR) is an active remote sensing system. Lidar echo signal is non-linear and non-stationary, which is often accompanied by various noises. In order to filter out the noise and extract valid signal information, a suitable method should be chosen for noise reduction. Some denoising methods are commonly used, such as the wavelet transform (WT), the empirical mode decomposition (EMD), the variational mode decomposition (VMD), and their improved algorithms. In this paper, a new denoising method named the WT-VMD joint algorithm based on the sparrow search algorithm (SSA), for lidar signal is selected by comparative experiment analysis. It is shown that this method is the most suitable one with the maximum signal-to-noise ratio (SNR), the minimum root-mean-square error (RMSE), and a relatively small indicator of smoothness when it is used in three kinds (50, 100, and 1000 pulses) of simulate lidar signals. The SNR is increased by 138.5%, 77.8% and 42.8% and the RMSE is decreased by 81.8%, 72.0% and 68.8% when being used to the three kinds of cumulative signal without pollution. Then, the SNR is increased by 83.3%, 60.4% and 24.0% and the RMSE is decreased by 70.8%, 66.0% and 50.5% when being used to the three kinds of cumulative signal with aerosol and clouds. The WT-VMD joint algorithm based on SSA is used in the denoising process for the actual lidar signal, showing extraordinary denoising effect and will improve the inversion accuracy of the lidar signal.

## 1. Introduction

As a combination of traditional radar technology and modern laser technology, lidar has the advantages of high spatial and temporal resolution, wide detection range, and so on [1]. It is an effective remote sensing tool to detect the vertical structure of the atmosphere [2,3], which is widely used in the measurement of atmospheric and environmental parameters such as cloud, aerosol, boundary layer, and visibility. In practical application, the intensity of lidar echo signal decreases gradually with the increase in detection distance, which is inversed to the square of detection distance. Moreover, due to the influence of solar background light, the dark current of the system photoelectric detector, the thermal noise of the amplifier, and other factors in the detection process, the weak echo signal at a long distance will be completely submerged in the noise. Due to the influence of various noises in the detection process, the echo signal at a long distance cannot obtain sufficient information [4]. The noise pollution of the lidar signal will affect the effective detection range and the accuracy of subsequent signal processing, so the noise reduction of lidar backscattered signal is an important research topic.

The noise in the lidar signal has the characteristics of continuous amplitude and random phase in time and can be regarded as a typical nonlinear and non-stationary signal. For this kind of signal processing, the current common denoising methods include wavelet transform (WT) [5], empirical mode decomposition (EMD) [6], and variational mode decomposition (VMD) [7]. Many experiments have demonstrated that these methods have significant effects on the removal of weak signal noise, and some studies have also applied these methods to the denoising experiments of lidar signals, showing that it also has a certain ability to deal with lidar signal noise [8,9,10]. For example, in 2004, the WT method was used to denoise lidar signals, increasing the effective detection range of lidar by Fang et al. [8]. EMD method is a signal processing method with strong adaptability, and it was used to denoise the lidar signal in 2009, which proved that the algorithm can effectively suppress the noise in the lidar signal and provide high SNR data for the subsequent inversion of extinction coefficient by Zhang et al. [9]. VMD method can make good use of its Vienna filtering characteristics to achieve a better smoothing effect. In 2018, Xu et al. compared and tested a variety of denoising methods, and successfully verified the excellent denoising performance of the VMD method in lidar echo signals [10].

With the deepening of denoising research, for the purpose of improving the performance of the single denoising algorithm, some scholars try to combine a variety of algorithms and have achieved specific research results [11,12]. Hu et al. pointed out that the combination of WT and the optimized VMD is able to perform the signal denoising and overcome the disadvantages of VMD and WT, whose performance is superior to the two individual methods [13]. In addition, Hua et al. proposed a denoising method to deal with the laser radar echo signal denoising problem based on the concept of joint denoising [14], in which they optimized the VMD parameters, including the number of decomposition modes and the quadratic penalty. In this paper, WT, EMD, VMD, and joint algorithms are selected to carry out noise filtering experiments to explore the performance differences of different methods for lidar signal noise, then choose the best denoising method for practical lidar signal processing. In order to solve the parameter selection problem of VMD, a swarm intelligence optimization algorithm, named the sparrow search algorithm (SSA) is used.

The rest of this paper is organized as follows. The related methods, including WT, EMD, VMD, WT-EMD, and the proposed denoising method WT-VMD, are introduced in Section 2. The simulation is presented in Section 3. In Section 4, the proposed method is compared with other existing methods, and the superiority of the proposed method is proved, then it is applied to measured load signals. Finally, discussions and conclusions are drawn in Section 5 and Section 6.

## 2. Method

### 2.1. Wavelet Transform: WT

The wavelet transform is based on Fourier transform and has good characteristics in the time-frequency domain. This method describes the signal as a superposition of a series of wavelet basis functions at different scales. The relationship between the frequency components and the wavelet basis functions can be characterized by the approximate coefficients and detail coefficients obtained after wavelet decomposition. The noise is contained in high-frequency components corresponding to the detail coefficients. Wavelet threshold denoising is the removal of signal noise by zeroing or shrinking the detail coefficients using a threshold function while retaining some of the approximate coefficients. The WT method requires the selection of a wavelet basis function that is similar to the signal to be decomposed. Appropriate decomposition level and threshold function can avoid incomplete separation of the signal or filtering out valuable information. It depends on the signal characteristics to determine whether the hard or soft threshold function is used. The hard thresholding function can preserve local features such as signal edges, but it is easy to cause distortion and discontinuity in the signal; the soft threshold function is relatively smooth but may suffer from loss of high-frequency information and blurred edges [15]. The selection of these parameters in advance will cause a difference in the denoising results [16,17]. The selection for the wavelet basis functions of WT will be introduced in Section 4.1.

### 2.2. Empirical Mode Decomposition: EMD

Empirical mode decomposition is an adaptive time-frequency processing method, which decomposes a set of nonstationary nonlinear signals into a series of intrinsic mode functions (IMFs) arranged from high to low frequency and a residual component (*res*).

(1) $$x(t)=\sum_{i=1}^{L}imf_{i}(t)+res(t),$$

where x(t) is the original signal, $imf_{i}(t)$ are the different IMF components, arranged according to the frequency, and res(t) is the residual quantity, which reflects the average trend of signal change.

Since the noise is primarily contained in the low-order modes (high-frequency components) of the IMFs, part of the high-frequency components can be directly discarded to achieve the purpose of denoising.

(2) $$\tilde{x}(t)=\sum_{i=k_{th}}^{L}imf_{i}(t)+res(t),$$

$\tilde{x}(t)$ is the reconstructed signal. Preserve the IMFs starting at $k_{th}$.

$k_{th}$ is determined by the correlation between the original signal x(t) and IMFs. The estimated ${\tilde{x}}_{m}(t)$ can then be rewritten as:

(3) $${\tilde{x}}_{m}(t)=\tilde{x}(t)=x(t)-\sum_{i=1}^{m}imf_{i}(t),$$

where $m=k_{th}-1$. The correlation coefficient between x(t) and $\tilde{x}(t)$ can then be calculated as follows:

(4) $$\rho (m)=\frac{\sum_{t=1}^{L}x(t)\tilde{x}(t)}{\sqrt{\sum_{t=1}^{L}x^{2}(t)\sum_{t=1}^{L}{\tilde{x}}^{2}(t)}},$$

in which ρ is always decreasing until it reaches a minimum. $k_{th}$ is given by [13]

(5) $$k_{th}=\arg\left\{\underset{1\leq m\leq L}{last}\right\}\left\{\rho (m)\geq C\right\}+1,$$

where m is the value at which ρ(m) starts to be smaller than some constant *C*. In general, *C* belongs to [0.75, 0.85] range.

In contrast to the WT method, the EMD method does not require a predetermined basis function and the appropriate mode function can be chosen to decompose the signal according to its own characteristics. Although the EMD method does not require the selection of parameters such as decomposition levels and threshold functions, modal confounding, endpoint effects, and difficulties in determining stopping conditions may occur [18]. Other denoisings, such as correlation-based EMD partial reconstruction (EMD-PR), EMD direct thresholding (EMD-DT), EMD interval thresholding (EMD-IT), and ensemble EMD (E-EMD) [19,20,21,22] denoising methods are often used for signal denoising, but there is no qualitative improvement for this study. In the future, we may make further detailed exploration and apply the improved EMD denoising algorithm to lidar signal processing.

### 2.3. Variational Mode Decomposition: VMD

Variational mode decomposition is also an adaptive signal processing method, which continuously updates each mode function $u_{k}$ and its center frequency by iteratively searching for the optimal solution of the variational modes, so as to decompose the noisy signal into a series of Band Limited IMFs (BLIMFs) with sparse and limited bandwidth [23].

(6) $$x\left(t\right)=\sum_{i=1}^{K}u_{i}\left(t\right),$$

where x(t) is also the original signal, and $u_{i}(t)$ represent different modes.

Contrary to EMD, the VMD method realizes the decomposition of the signal from low frequency to high frequency. The noise is hidden in the high-order BLIMFs. Therefore, the VMD method selects the low-order modal function to reconstruct directly to remove the noise.

(7) $$\tilde{x}\left(t\right)=\sum_{i=1}^{k_{{}_{th}}}u_{i}\left(t\right),$$

Compared with the EMD method, it has a fast convergence speed, strong robustness and can effectively avoid problems such as modal aliasing and end effects. However, VMD is not suitable for all non-stationary signals. Some non-stationary signals need to be preprocessed before using VMD.

When VMD is used for signal decomposition, the number of decomposition layers *K* and the secondary penalty factor α needs to be set, of which the numerical selection has a great influence on the decomposition results [24,25]. Usually, the parameters of *K* and α are empirically set according to the signal characteristics. In order to accurately display the performance of the VMD algorithm, it is necessary to search for the optimal values of *K* and α.

However, in practice, manual search is not feasible. Consider using swarm intelligence optimization algorithms to apply to VMD parameter selection problems. Sparrow search algorithm (SSA) is an effective optimization technique, which simulates the foraging and anti-predation behaviors of sparrows [26]. Its parameters are few, adaptable, convergence accuracy performance is good, and with strong global search capabilities, for single-modal and bimodal functions can obtain the best optimal solution and average solution.

The fitness function in SSA needs to be determined according to the function characteristics of the parameters to be optimized. According to the VMD algorithm of the parameters to be optimized in this paper, the energy entropy is selected as the fitness function. Energy entropy reflects the distribution of signal energy in the time domain, and its size is inversely proportional to the proportion of the signal in the total energy. In the process of parameter combination optimization, it is necessary to calculate the energy entropy of each $u_{k}$, and the formula is as follows:

(8) $$H=-\sum_{k=1}^{K}p(k)\log p(k),$$

where $p(k)=E_{k}/E$ is the proportion of the energy of each IMF in the total signal energy; $E_{k}=\sum u_{k}{\left(t\right)}^{2}$, $E=\sum E_{k}$.

The local minimum energy entropy is used as the *fitness* function in SSA. The formula is as follows:

(9) $$fitness=\underset{\gamma =(K,\alpha )}{\min}\left\{\min_{L},H_{imf}\right\},$$

### 2.4. Joint Algorithm

The single denoising algorithm has its own merits and demerits. Both EMD and VMD achieve efficient denoising by directly removing high-frequency modes, but there is also a small amount of signal information mixed in the high-frequency components. WT processes the noise components by threshold so as to protect useful signal peaks and bursts well, but it also leads to incomplete noise removal. Therefore, this paper attempts to combine WT and EMD/VMD algorithms to enhance the denoising ability and obtain more efficient results.

WT-EMD: first, the raw noisy signal is denoised by WT, then the denoised signal is decomposed using EMD. Finally, the signal is reconstructed by superposition of the relevant components.

WT-VMD: after the overall denoising process with WT, the denoised signal is further decomposed by the VMD method and then the relevant BLIMFs are selected for reconstruction to complete the secondary denoising.

## 3. Simulation

**Figure 1:**
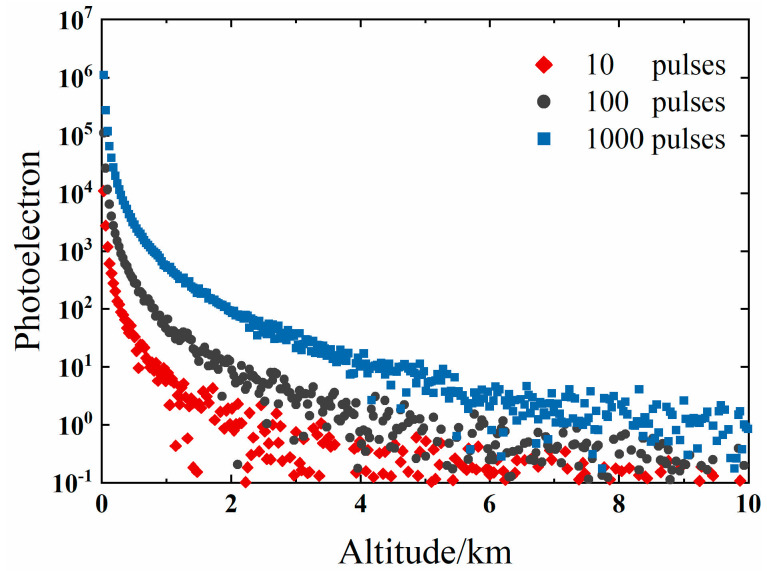
Simulation results of cumulative pulse signals with noise.

The echo signal received by lidar in the actual work can be simulated by simulating the number of ideal lidar echo photoelectrons and adding Poisson noise [27,28]. Due to the weak intensity of the single pulse-echo signal, the cumulative average of multiple pulses is usually used to enhance the signal-to-noise ratio (SNR) in the practical application of lidar. The higher the number of accumulated pulses, the lower the proportion of noise component in the signal. Figure 1 shows the simulation results of the photoelectron number distribution of lidar signal with different cumulative pulse times (10 pulses, 100 pulses, and 1000 pulses). Raising the cumulative number of pulses can effectively raise the detection range of lidar echo signals. Within the same detection range, the signal-to-noise ratio continues to increase as the number of pulses increases.

## 4. Experiments

### 4.1. Parameter Selection of Denoising Method

The wavelet basis functions of WT include haar, sym, coifle, dB, etc. In this paper, the sym wavelet is chosen as the wavelet basis function. Sym wavelet is a typical discrete wavelet basis with good orthogonality and symmetry, which is suitable for the processing of lidar echo signals. The vanishing moment sym3–sym8 and decomposition level 3–6 are chosen, respectively, to denoise the simulation echo signal. It is experimentally determined that the denoised signal has the best SNR and the lowest RMSE when the wavelet basis function is sym7 and the decomposition level is 4. Therefore, this paper uses the sym7–4 scheme for wavelet denoising in the subsequent experiments. In order to obtain better overall continuity of the signal, a soft threshold function is chosen for denoising in this paper.

The selection of EMD relevant components and VMD decomposition levels can be determined adaptively by using the methods mentioned in Section 2.2 and Section 2.3.

### 4.2. Evaluation Indicators of Denoising

The noise of lidar signal is filtered by different single algorithms and combined algorithms, respectively, and the denoising effects are evaluated by three indicators: root mean square error (RMSE), signal-to-noise ratio (SNR), and smoothness (*r*) [29]:

(10) $$SNR=10\times \mathrm{lg}\left(\frac{\sum_{n}f^{2}\left(n\right)}{\sum_{n}{\left(f^{\prime }\left(n\right)-f\left(n\right)\right)}^{2}}\right),$$

(11) $$RMSE=\sqrt{\frac{1}{n}\underset{n}{\sum }{\left(f^{\prime }\left(n\right)-f\left(n\right)\right)}^{2}},$$

(12) $$r=\frac{\sum_{n-1}{\left(f^{\prime }\left(n+1\right)-f^{\prime }\left(n\right)\right)}^{2}}{\sum_{n-1}{\left(f\left(n+1\right)-f\left(n\right)\right)}^{2}},$$

where f(n) is the raw signal before denoising, and $f^{\prime }(n)$ is the output signal after denoising. The higher *SNR* means the lower noise ratio. The smaller *RMSE* means closer to the raw clean signal. The smaller *r* means the smoother curve.

### 4.3. Simulation Signals with No Pollution

**Figure 2:**
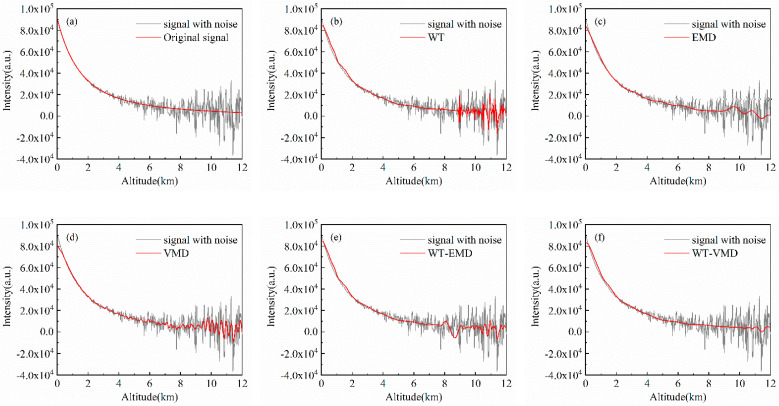
Experimental results of denoising of 50-pulse cumulative simulation signals (no pollution): (**a**) original signal; (**b**) WT; (**c**) EMD; (**d**) VMD; (**e**) WT-EMD; (**f**) WT-VMD.

**Figure 3:**
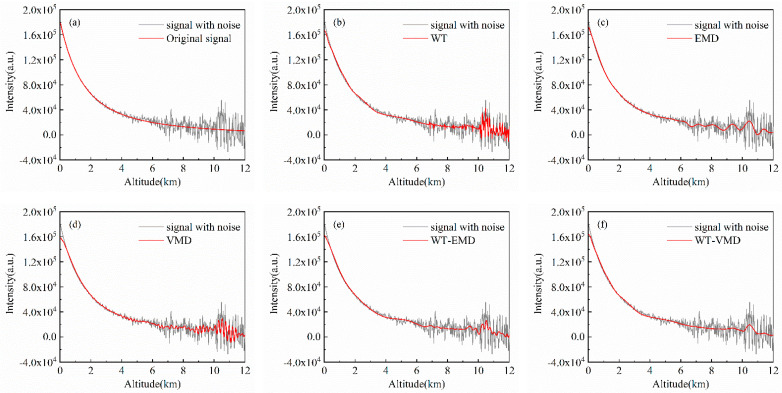
Experimental results of denoising of 100-pulse cumulative simulation signals (no pollution): (**a**) original signal; (**b**) WT; (**c**) EMD; (**d**) VMD; (**e**) WT-EMD; (**f**) WT-VMD.

**Figure 4:**
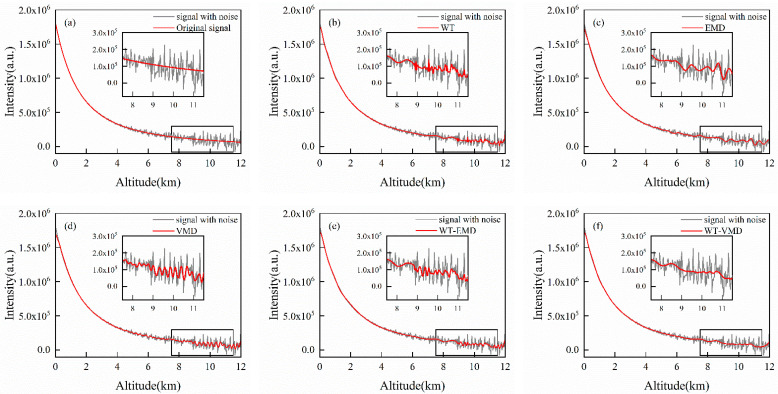
Experimental results of denoising of 1000-pulse cumulative simulation signals (no pollution): (**a**) original signal; (**b**) WT; (**c**) EMD; (**d**) VMD; (**e**) WT-EMD; (**f**) WT-VMD.

A clear sky aerosol model is used to simulate lidar signals without pollution, and three sets of different cumulative pulse numbers (50\100\1000) represent different original SNR conditions. Denoising experiments are carried out using the five different denoising algorithms mentioned above, and the results are shown in Figure 2, Figure 3 and Figure 4 and Table 1, Table 2 and Table 3, respectively.

The original clean signal without air pollution is a smooth curve. Due to the large amount of noise in the remote distance, the simulation noisy signal fluctuates greatly with the bad smoothness of the curve. The signal noise content of 50 accumulated pulses is relatively high with the original input SNR of 10.6707dB. As the number of cumulative pulses increases, the input SNR of 1000 cumulative pulse signals can reach 23.6621 dB, and the smoothness decreases significantly. In the denoising experiments, despite all algorithms filtering out noise to a certain extent, there is variability in the results of different denoising methods under different input signal-to-noise ratio conditions. The effect of WT denoising method is not sufficiently thorough, there still remain large signal fluctuations in the remote distance after denoising. However, when the number of cumulative pulses increases, the output SNR of WT increases greatly. Under the condition of high noise, decomposing the signal with EMD and retaining the low-frequency components allow for maximum noise filtering, resulting in better denoising. Retaining only the low-frequency components ensures the smoothness of the signal so that the EMD maintains a good advantage in pollution-free atmospheres with different SNRs. The denoising capability of VMD is relatively stable and does not change significantly with different input SNRs, while the VMD-processed signal deviates more from raw signal at the near-end points. The WT-EMD joint algorithm improves the denoising effect of WT, but it does not perform as well as the EMD algorithm at high noise levels. In all three groups of experiments, WT-VMD has the best denoising effect which can reduce the smoothness of the denoised curve to below 1. In the case of 50 cumulative pulse signals, the signal denoised by WT-VMD can improve the output SNR to near 2.5 times the original SNR. From Table 1, Table 2 and Table 3, we can see that the SNR is increased by 138.5%, 77.8%, and 42.8%, and the RMSE is decreased by 81.8%, 72.0%, and 68.8% when the proposed method is used for the three kinds of cumulative signal without pollution.

**Figure 5:**
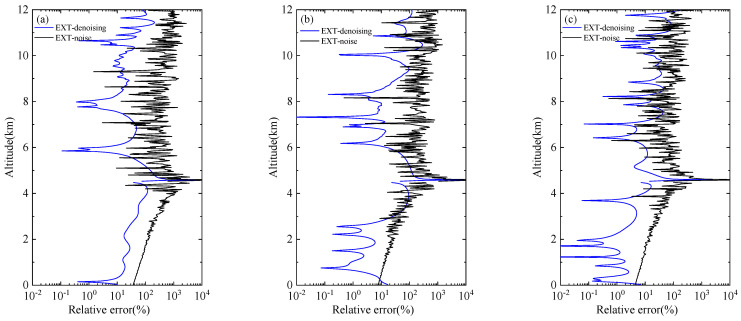
Comparison of relative error of extinction coefficient before and after denoising (no pollution): (**a**) 50-pulse cumulative simulation signals; (**b**) 100-pulse cumulative simulation signals; (**c**) 1000-pulse cumulative simulation signals.

The ultimate goal of lidar signal denoising is to retrieve the real distribution of the atmosphere better, so it is necessary to further verify whether the denoised signal can improve the accuracy of subsequent inversion. The Fernald method [30,31] is used to invert the original clean signal, the noise signal, and the WT-VMD denoised signal, respectively. The black line in Figure 5 shows the relative error between the extinction coefficient of the noise signal and that of the clean signal at different SNRs, and the blue line means the relative error between the extinction coefficient of the denoise signal and that of the clean signal at different SNRs. When the number of cumulative pulses is small, that is the noise content is relatively high, and the error of extinction coefficient of undenoised signal is large. The result of 50 cumulative pulses is an order of magnitude greater than the error of 1000 cumulative pulses. The three experimental results all prove that the extinction coefficient obtained after denoising is closer to the real value.

### 4.4. Simulation Signals with Aerosol Layers and Clouds

**Figure 6:**
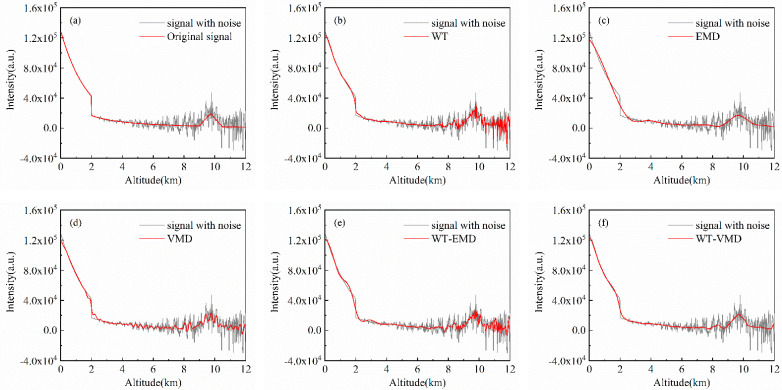
Experimental results of denoising of 50-pulse cumulative simulation signals (with aerosol and clouds): (**a**) original signal; (**b**) WT; (**c**) EMD; (**d**) VMD; (**e**) WT-EMD; (**f**) WT-VMD.

**Figure 7:**
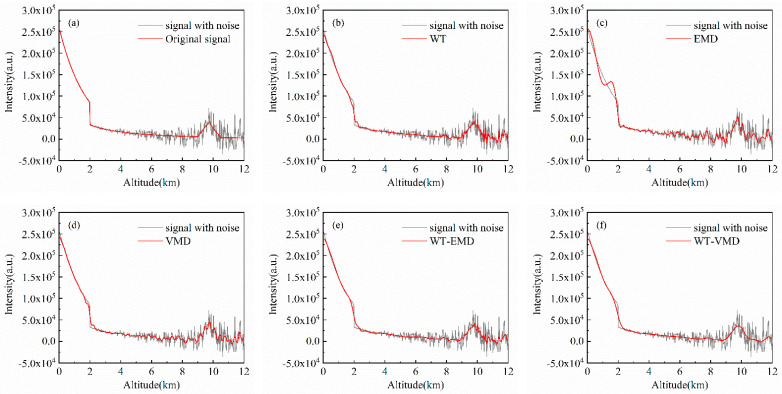
Experimental results of denoising of 100-pulse cumulative simulation signals (with aerosol and clouds): (**a**) original signal; (**b**) WT; (**c**) EMD; (**d**) VMD; (**e**) WT-EMD; (**f**) WT-VMD.

**Figure 8:**
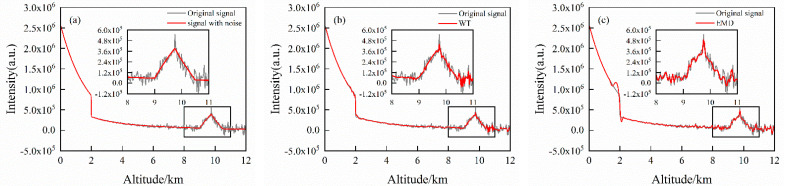

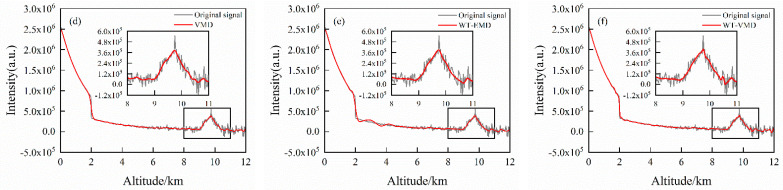
Experimental results of denoising of 1000-pulse cumulative simulation signals (with aerosol and clouds): (**a**) original signal; (**b**) WT; (**c**) EMD; (**d**) VMD; (**e**) WT-EMD; (**f**) WT-VMD.

To demonstrate the capability of these methods can be applied to wide conditions, this paper also simulates signals with realistic aerosol (0–2.5 km) and cloud distributions (9.5–11.5 km). The lidar echo signal with aerosol layer and clouds is not a simple smooth curve. These denoising methods should be tested that if they can retain the information of aerosol and cloud while denoising. Under this simulated condition, three groups of denoising experiments with different cumulative pulse numbers are also carried out. The results show in Figure 6, Figure 7 and Figure 8 and Table 4, Table 5 and Table 6, respectively.

WT can retain more signal characteristics, especially good expression of the aerosol layer structure. However, also because of this, WT retains more noise resulting in a poor denoising effect under the condition of low SNR. EMD method pursues high smoothness of curve, which will weaken signal properties such as aerosol layer leading to large deviation from the original signal. It is apparent that this situation is more likely to happen when the signal has a low content of noise. For example, the aerosol layer of the signal after EMD denoising has a deviation in the experiment of 1000 cumulative pulse signals. Signals processed by VMD also retain part of the fluctuation with larger smoothness, but better than that after WT denoising. In the experiment of 50 cumulative pulse signal denoising, the VMD method still has the problem of endpoint deviation. Similar to the experimental results of denoising in the case of no pollution, when the cumulative pulse signal is 50 or 100 times, the results of WT-EMD are slightly better than that of the single WT method. When the cumulative pulse number reaches 1000 times, the RMSE of the WT-EMD combined method increase. Because the WT method performs well under high SNR and can filter out a large amount of noise while EMD is prone to modal aliasing under high SNR conditions. Therefore, the combined algorithm (using EMD secondary denoising again on the basis of WT) may perform worse. The WT-VMD method is still the best in the denoising experiment for the lidar noise signal with aerosol and clouds. Compared with the original signal, the results not only ensure good smoothness but also show the aerosol and cloud structure well. In addition, the WT-VMD joint algorithm improves the endpoint problem of a single method VMD. It is noted that we can see that the SNR is increased by 83.3%, 60.4%, and 24.0%, and the RMSE is decreased by 70.8%, 66.0%, and 50.5% when the proposed method is used for the three kinds of cumulative signal with aerosol and clouds from Table 4, Table 5 and Table 6.

**Figure 9:**
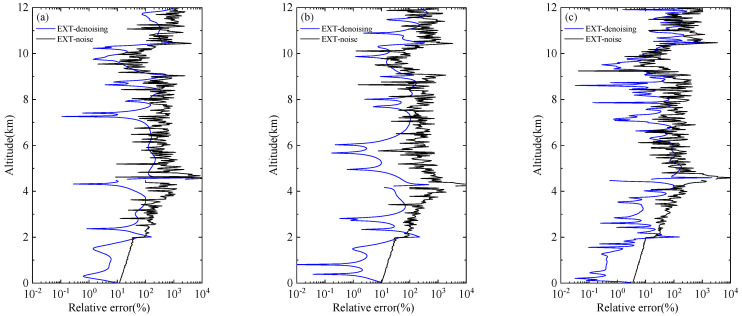
Comparison of relative error of extinction coefficient before and after denoising (with aerosol and clouds): (**a**) 50-pulse cumulative simulation signals; (**b**) 100-pulse cumulative simulation signals; (**c**) 1000-pulse cumulative simulation signals.

The results of extinction coefficient before and after signal denoising under cloud and aerosol conditions are also compared in Figure 9. The inversion error of 1000 cumulative pulses signal is obviously less than 50 and 100 cumulative pulses signal. After denoising, the relative errors of the three extinction coefficients can reach a similar level. It is noticed that the relative error decreases obviously after denoising at 0–2 km aerosol layer and 9.5–11.5 cloud layer. This indicates that the WT-VMD method has a significant effect on improving the inversion accuracy of aerosol and cloud structure.

### 4.5. Denoising Results with Real Lidar Signals

**Figure 10:**
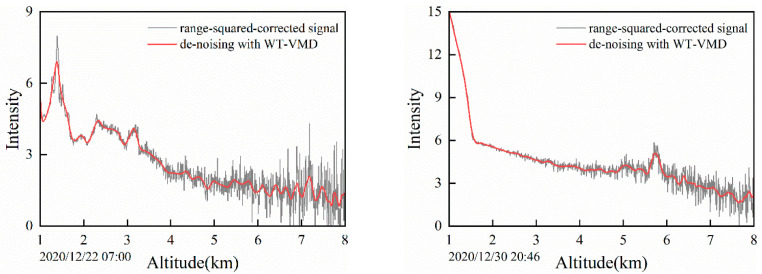
Comparison of denoising results for range-squared corrected signal.

It is necessary to verify the performance of the WT-VMD method by experimenting on the actual lidar echo signal, which is complicated by various interferences. As the altitude increases, the power of the lidar signal attenuates with the square of the distance. In general, the range-square corrected signal is obtained to eliminate the distance’s influence on the echo signal by multiplying the square of the distance by the echo power. The WT-VMD method is used to filter the signals’ noise when selecting the rang-square corrected signals detected by lidar at two different times in Hefei. As shown in Figure 10, when the detection distance is 4 km, the signal is gradually submerged by noise and no useful information can be obtained. While after processing by the WT-VMD, the effective detection range can be increased to more than 6 km. It shows that the WT-VMD method can make the weak signal easier to detect while maintaining the original signal structure. For the lidar data detected at different times, the WT-VMD method has a strong ability to deal with the noise.

**Figure 11:**
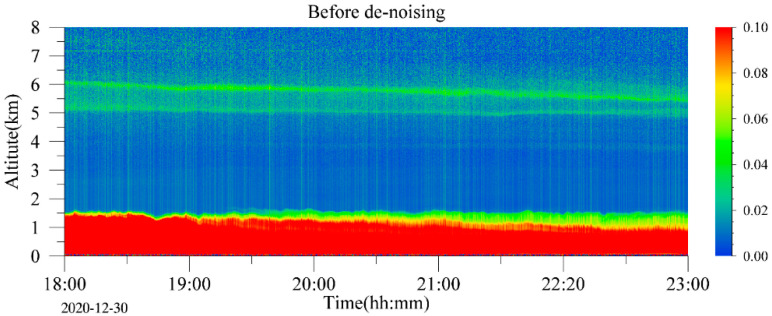

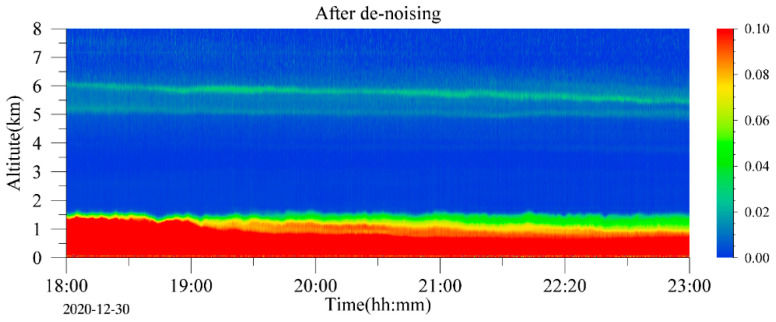
Comparison of aerosol extinction coefficient before and after denoising.

To intuitively illustrate the influence of noise on the result of signal inversion, selecting the lidar signal data, which is averaged using 100 cumulative pulses for each profile within 10 s and its sampling rate is 40 MHz, measured in Heifei from 18:00 to 23:00 on 30 December 2020. The range-squared corrected signals were inverted by the Fernald method after the WT-VMD denoising. Extinction coefficient results of lidar signal inversion before and after denoising are shown in Figure 11. The noise in the original lidar signal will cause the error of the inversion results. There are strange vertical lines and a lot of noise points at long distances in the spatio-temporal diagram of the extinction coefficient before denoising. After denoising, the long-distance noises are removed, and the extinction coefficient image shows the structure of the near-distance aerosol layer more clearly. The experimental result proves that the WT-VMD method can suppress the high-frequency peak noise and improve the inversion accuracy effectively.

### 4.6. Comparison with Averaging Signals

In daily lidar signal processing, time averaging is often used as an effective denoising method. The longer the average time, the higher the SNR. In order to compare the difference between WT-VMD method and time averaging method, the one-hour lidar signal measured in Heifei from 15:00 to 16:00 on 8 September 2021 is selected as experimental data.

**Figure 12:**
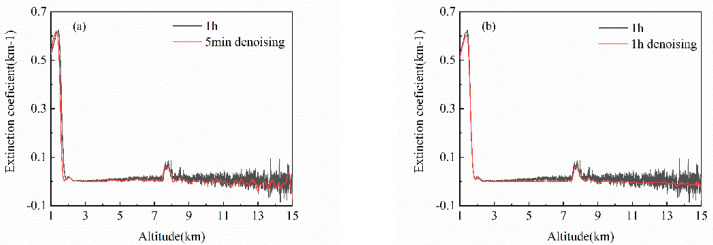
Comparison of extinction coefficient between averaging method and WT-VMD method (**a**) 1 h averaging vs. 5 min WT-VMD denoising; (**b**) 1 h averaging vs. 1 h WT-VMD denoising.

The one-hour lidar signal is cumulative averaged and compared with the 5 min signal denoised by WT-VMD. The Figure 12a shows the extinction coefficient results in both cases. The 1 h averaging signal still had a lot of noise after 9 km, while the noise content of the 5 min signal decreased significantly after using WT-VMD. Figure 12b presents the extinction coefficients of 1 h averaging signal before and after denoising. Using WT-VMD to denoise the one-hour averaging signal makes the signal smoother and the signal structure clearer. The experiment demonstrates that the averaging method can only reduce noise to a certain extent, but its noise filtering effect is far less than that of WT-VMD. Atmospheric changes are instantaneous. Using too long averaging time to process signals will hide the true changes in atmospheric distribution. Therefore, the one-hour averaging signal is not suitable for real lidar signal procession. While the time averaging signal shorter than 1 h will have more noise, which is definitely worse than the signal denoised by the WT-VMD method.

**Figure 13:**
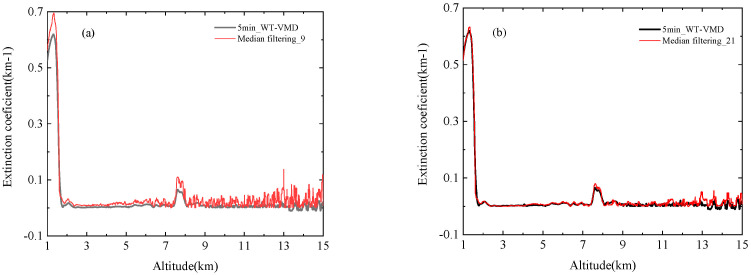
Comparison of extinction coefficient between median filtering method and WT-VMD method (**a**) median filtering_9 vs. 5 min WT-VMD denoising; (**b**) median filtering_21 vs. 5 min WT-VMD denoising.

Median filtering is a signal processing method based on sorting theory, which can suppress impulse noise well. The main principle is to replace a point in the number sequence with the median value of other neighboring data points, so that the value of the point is closer to the real value. This method can eliminate isolated data points and protect the edge of signal from being blurred. It is also commonly used in practical signal processing. A lidar signal profile measured in Hefei at 15:10 on 8 September 2021 is selected as experimental data. Figure 13 shows the comparison result of the 5 min signal after denoising of the WT-VMD and the signal after median filtering. In Figure 13a, the filtering is not complete when L = 9 is chosen as the window length, the noise fluctuates significantly at altitudes above 8 km, and the extinction coefficients of the inversion at close distances show an overall deviation, which is the inversion error caused by the insufficient signal-to-noise ratio. When the window length is increased to 21, as shown in Figure 13b, the noise fluctuations in the upper layers are reduced, but the noise filtering effect is still not as good as the WT-VMD denoising algorithm. The choice of window values determines the smoothing quality, and the longer the window, the better the smoothing effect. If the window is too small and the length is less than nine, the denoising effect must be worse. In order to improve the denoising performance, if you select a window with a length greater than 21, it reduces the spatial resolution of the signal and blurs the atmospheric structure.

## 5. Discussions

When the number of cumulative pulses is large, that is, the SNR of the original signal is high, all kinds of denoising methods can effectively remove most of the noise information. However, when the cumulative pulse decreases, that is, the SNR decreases, and the difference of noise reduction ability among different methods increases.

From the perspective of denoising evaluation indicators, the related methods of VMD are better than the related methods of EMD in terms of SNR, RMSE or smoothness. Among them, the proposed WT-VMD method has absolute advantages, and it has the best noise reduction performance under different sizes of original SNR, and its advantages are more obvious when the noise is more.

The effect of WT is the worst. The overall wavelet threshold denoising can only weaken the protrusion of the curve, but there is still a lot of noise information left, and the denoising is not complete. Additionally, with the increase in noise, the shortage of the WT method in filtering noise is becoming more and more prominent. Although the smoothness of the curve obtained by the EMD method is small, the endpoint effect may occur when the SNR is low, that is, the signal endpoint offset is large, causing signal distortion, and modal aliasing is easy to occur when the SNR is high.

As to the joint denoising, the filtering ability of WT-EMD and WT-VMD has been significantly improved. However, not all joint denoising methods are significantly better than the single denoising method. Since there are still useful signals hidden in high-frequency components after EMD/VMD decomposition, WT processing for low-frequency components with less noise information cannot achieve the purpose of secondary effective denoising. On the contrary, if WT is used to filter most of the noise, then EMD/VMD decomposition. At this time, the noise content in the high-frequency component is higher. After discarding the high-frequency component, the SNR can be improved more.

## 6. Conclusions

In order to find the most suitable denoising method for lidar signals, this study employs the joint algorithms WT-EMD and WT-VMD based on the single denoising algorithm WT, EMD, and VMD. The comparative experiment of denoising is carried out by using a simulation lidar signal. The simulated signal with noise is divided into two types, one is under the condition of no pollution, and the other is with aerosol and cloud distribution. Three kinds of experiments were conducted with 50, 100, and 1000 cumulative pulses, respectively, corresponding to different input SNR. Three indicators, SNR, RMSE, and smoothness, are used to evaluate the noise reduction effect of the algorithm.

The experimental results show the difference in denoising ability under different denoising methods. WT method is more suitable for the condition of high SNR and can restore the signal structure well, but the signal after denoising still has a lot of fluctuations in the far distance and the smoothness of the curve is poor. EMD method performs well in pollution-free weather, but it will lose the details of atmospheric distribution for aerosol and clouds. VMD method has stable denoising capability under different noise levels. Its overall effect is better than WT, but there may exist an endpoint problem. Joint algorithms are usually superior to single algorithms. WT denoising can filter out most of the noise while retaining the signal characteristics, and then EMD/VMD can further remove the remaining noise in detail. However, mode aliasing may occur in EMD at high SNR, so WT-EMD sometimes leads to signal distortion. The result indicates that the WT-VMD method has the best noise reduction effect. Regardless of the noise level, the signal processed by it has the minimum RMSE, the maximum SNR, and a relatively small indicator of smoothness. Then, the denoised signal by WT-VMD based on SSA can display the information of the original aerosol and cloud well and reduce the relative error of the extinction coefficient obviously.

Applying the proposed method to the actual lidar data can effectively reduce the signal’s noise with the increasing detection range and enhance the accuracy of subsequent aerosol extinction coefficient inversion. Compared with the common averaging method and the median filtering method, the atmospheric inversion results obtained by WT-VMD denoising are also better. The experimental results demonstrated that the WT-VMD method based on SSA could be well used in practical lidar signal processing. In the next step, the algorithm will be further optimized to improve the operation speed, so as to play a greater role in the actual lidar atmospheric detection.

## Figures and Tables

**Table 1:** Evaluation Index of 50-pulse cumulative signal (no pollution) after denoising.

Method	SNR/dB	RMSE	r
Original signal	10.6707	7.6350 × 10^3^	763.6516
WT	18.4471	3.1188 × 10^3^	99.8146
EMD	23.4080	1.9081 × 10^3^	0.8941
VMD	24.7626	1.5073 × 10^3^	0.8715
WT-EMD	19.4122	2.7908 × 10^3^	4.9110
WT-VMD	25.4534	1.3921 × 10^3^	0.8074

**Table 2:** Evaluation index of 100-pulse cumulative signal (no pollution) after denoising.

Method	SNR/dB	RMSE	r
Original signal	14.2068	1.0163 × 10^4^	311.3069
WT	20.8722	4.7181 × 10^3^	47.4279
EMD	23.9733	3.3015 × 10^3^	1.1584
VMD	23.6670	3.4200 × 10^3^	2.1083
WT-EMD	23.4743	3.4967 × 10^3^	1.9071
WT-VMD	25.2559	2.8483 × 10^3^	0.8202

**Table 3:** Evaluation index of 1000-pulse cumulative signal (no pollution) after denoising.

Method	SNR/dB	RMSE	r
Original signal	23.6621	3.4219 × 10^4^	33.5717
WT	31.7819	1.3436 × 10^4^	2.6689
EMD	31.5026	1.3875 × 10^4^	0.9959
VMD	30.5857	1.5420 × 10^4^	1.5345
WT-EMD	31.6379	1.3661 × 10^4^	1.5550
WT-VMD	33.7913	1.0661 × 10^4^	0.9061

**Table 4:** Evaluation index of 50-pulse cumulative signal (with aerosol and clouds) after denoising.

Method	SNR/dB	RMSE	r
Original signal	12.8423	7.7208 × 10^3^	63.6080
WT	18.7713	3.9013 × 10^3^	12.3046
EMD	20.3827	3.2407 × 10^3^	0.1751
VMD	21.6894	2.7898 × 10^3^	0.4344
WT-EMD	19.3723	3.6405 × 10^3^	2.4556
WT-VMD	23.5406	2.2529 × 10^3^	0.2511

**Table 5:** Evaluation index of 100-pulse cumulative signal (with aerosol and clouds) after denoising.

Method	SNR/dB	RMSE	r
Original signal	15.5153	1.1351 × 10^4^	30.0035
WT	23.0867	4.7421 × 10^3^	2.2018
EMD	18.4324	8.1141 × 10^3^	1.0202
VMD	24.6126	3.9827 × 10^3^	0.3910
WT-EMD	23.8689	4.3387 × 10^3^	0.6280
WT-VMD	24.8893	3.8578 × 10^3^	0.2619

**Table 6:** Evaluation index of 1000-pulse cumulative signal (with aerosol and clouds) after denoising.

Method	SNR/dB	RMSE	r
Original signal	25.4364	3.6223 × 10^4^	4.3180
WT	31.4271	1.8174 × 10^4^	1.0682
EMD	24.9903	3.8132 × 10^4^	0.9416
VMD	29.8152	2.1880 × 10^4^	0.4904
WT-EMD	27.9345	2.7169 × 10^4^	0.3050
WT-VMD	31.5388	1.7942 × 10^4^	0.4273

## Data Availability

Data underlying the results presented in this paper are available by contacting the first author or the corresponding author.
